# A Review of Academic Use of the Term “Minor Attracted Persons”

**DOI:** 10.1177/15248380241270028

**Published:** 2024-09-15

**Authors:** Christina Farmer, Michael Salter, Delanie Woodlock

**Affiliations:** 1 University of New South Wales, Sydney, Australia; 2 Monash University, Clayton, VIC, Australia

**Keywords:** child sexual abuse, pedophilia, minor attraction, prevention, treatment

## Abstract

Although it originated within online pro-pedophile groups, the term “minor attracted person” (MAPs) has been adopted by some academic researchers as a neutral and non-stigmatizing alternative to the term “pedophile.” The transferral of this term from pedophile advocates to academic scholarship has been highly controversial. Claims that the use of the term “minor attracted people” normalizes or endorses pedophilia deserve closer scrutiny. This paper is based on a rapid evidence review of all peer-reviewed papers between 2015 and 2023 that used variants of the term “minor attracted” in their title and/or abstract. After screening, 30 studies were identified for review. Our analysis took a thematic approach to understanding the construction and use of the term MAPs in this scholarship. The analysis found that the term MAPs was operationalized in different and contradictory ways, however, the literature broadly agreed that MAPs constitute an oppressed sexual minority who are subject to undue stigmatization and discrimination. We point to the similarities between this sympathetic framing of MAPs and the political goals of the pro-pedophile advocacy groups that created the term MAPs, and from which many MAPs studies recruit their research participants. The review concludes that, in the absence of adequate self-reflexivity and awareness of bias, academic collaborations with pro-pedophile groups can produce work that minimizes the risk and harm of child sexual abuse and has the potential to delegitimize child sexual abuse prevention and treatment efforts.

## Critical Findings

- The use of the term MAPs has been adopted by a small group of academics in the fields of child sexual abuse prevention and the clinical treatment of pedophilia.
- MAPs is defined in this literature in various and sometimes contradictory ways, including as a category for any person with sexual feelings toward children, as a synonym for pedophilia, or as a sexual identity, orientation, or minority.
- Academic MAPs scholarship does not openly acknowledge the origin of the term “MAPs” in online pro-pedophile organizations; however, academic use of the term contains many of the same assumptions and arguments put forth by those organizations.
- MAPs scholarship presents pedophiles as an oppressed sexual minority subject to undue discrimination and oppression. This framing draws, implicitly and explicitly, on controversial comparisons between pedophilia and same-sex attraction.
- Strong claims in the MAPs literature that the stigmatization of sexual interest in children is the primary driver of child sexual abuse, and thus sexual interest in children should be socially and culturally normalized, are empirically unsupported and contrary to child protection prerogatives.
- Many of the assertions contained in academic scholarship using the term MAPs are congruent with the long-standing political goals of pro-pedophile advocates and activists.

## Implications for Research, Policy, and Practice

- “MAPs” is part of the nomenclature of contemporary pro-pedophile movements seeking social and legal rights for those with a sexual interest in children and is not a neutral or scientific synonym for pedophile.
- Pedophile movements and groups have conflicted, dissembling, and sometimes positive views about the sexual abuse of children, and therefore academic engagement with these movements should be cautious and informed.
- Research studies based on the recruitment of research subjects from online pedophile networks and spaces must acknowledge, as an urgent matter of ethical and research integrity, the likelihood of bias.
- Research into undetected pedophiles and offenders in the community should consider methodological approaches that do not depend on support or sponsorship by online pedophile networks.
- The stigmatization of the impulse to sexually abuse children plays an important role in deterring child sexual abuse and delineating between acceptable and unacceptable sexual behavior.
- Academic comparisons between pedophilia and same-sex attraction run the risk of undermining LGBTIQ+ civil rights and delegitimizing the project of child sexual abuse prevention.

## Introduction

Over the last several years, the use of the term “minor attracted people” (MAPs) as an alternative to “pedophile” has migrated from paedophile^1^ advocacy groups into scientific and clinical literature. Scholars who use the term MAPs claim it is a neutral, non-stigmatizing, and more accurate way of referring to people who are sexually interested in children. However, public response to this development has been overwhelmingly negative. Critics of the term are concerned that it normalizes pedophilia (Svrluga, 2021), trivializes the impacts of child sexual abuse, and conflates sexual attraction to children with same-sex attraction (Bindel, 2023). Journalists have noted close associations between scholars who use the term MAPs and pro-pedophile organizations who seek greater visibility and acceptance of sexual interest in children (Bindel, 2023). Controversy over the scholarly use of the term MAPs has occurred against a backdrop of scandal over academic papers that have been accused of endorsing child sexual abuse and other deviant sexual behavior.^2^

It might be tempting to dismiss allegations of academic toleration of pedophilia as little more than a moral panic. Indeed, those academics whose work has been bought into question have claimed to be the target of discrimination and right-wing opprobrium. It is certainly the case that right-wing social media accounts, tabloids, and television stations have played an outsized role in bringing these issues to public attention. At the same time, there is reason for caution. As this article will detail, the history of academic engagement with pro-pedophile groups has been fraught with conflicts of interest (Goode, 2011). Such work has legitimized gross failures of child protection such as the deliberate placement of foster children in the homes of known pedophiles in Germany (Aviv, 2021), state support for pro-pedophile advocacy in England (Pilgrim, 2018), as well as sustained efforts to delimit child protection and criminal justice intervention in sexual abuse in the United States (Goode, 2011).

It is therefore important that close attention and critical scrutiny is paid to the transferral of terminology and argumentation from pro-pedophile groups into academic literature. After providing a brief history of the intersections between pedophile groups and scholarly research into child sexual abuse, this article presents a rapid evidence review of scholarship that has utilized the terminology of MAPs. The review seeks to understand how this body of scholarship defines and uses the terminology of MAP, conceptualizes sexual interest in minors, and its relationship with child sex offending. and the implications for child protection and safeguarding. The review identified 30 articles published between 2015 and 2023, and findings are arranged under three overarching themes: (a) definitions of the term MAPs, (b) “MAPs” as an oppressed sexual minority, and (c) recommendations for the de-stigmatization of sexual interest in children. In our analysis, we point to areas of conceptual confusion in this literature, including the simultaneous denial that MAPs pose a risk to children while insisting that they are at risk of offending if they are stigmatized. We argue that such contradictions are unsustainable and have significant child protection implications. We also raise concern about the ethics of the comparisons between pedophilia and same-sex attraction that are evident throughout MAPS scholarship. The findings of the review suggest that the terminology of MAPS remains linked to the agenda of social movements of pedophiles and calls for critical analysis of scholarly cooperation and engagement with pro-pedophile advocacy.

## The History of the Term “MAPs”

As Goode (2009, p. 62) notes, a particular challenge facing researchers seeking to study men in the community with a sexual interest in children (rather than child sex offenders in treatment or prison) is that they are often reliant on “the goodwill of the ‘pedophile community’ to advertise the project and provide data.” Advocacy groups of self-identified pedophiles have been active in the United States and Europe since the mid-20th century with the goal of promoting child sexual abuse and abolishing the age of consent (DeYoung, 1989). Almost since their inception, researchers have been engaging with these groups to study men with a sexual interest in children who have not been caught or convicted of an offense. The work of foundational sexologist Alfred Kinsey included extracts taken from the diary of at least one prolific child sex offender, which were used to justify Kinsey’s claim that child sexual abuse by adults is harmless or beneficial (Goode, 2011). In interview, Kinsey’s coauthor Paul Gebhard described undertaking interviews with an American pedophile network as part of this research program (Tate, 1998). Since Kinsey’s time, scholars who collaborate with pro-pedophile movements have produced a range of studies claiming that sexual contact between adults and children can be harmless or beneficial for children and society (Dallam, 2002; Pilgrim, 2018).

Today, pro-pedophile organizations flourish online and continue to be engaged by researchers with varying levels of critical reflection on how the norms and political commitments of these groups may influence the response of their members to research questions (Goode, 2009, 2011). The recent academic adoption of the terminology of “MAPs” is one such example. From the late 1990s, online communities and networks of pedophiles began agitating for the use of the term “MAPs” as a non-stigmatizing and more inclusive alternative to “pedophilia.” The use of term “minor attracted” appears to have been coined in 1998 on the “Boychat” messageboard, which, according to the webmasters who ran the site, catered to men with “a particularly affinity for teen and/or pre-teen boys” (Malesky Jr & Ennis, 2004, p. 94). Boychat included an active cohort of users promoting the sexual abuse of children. An analysis of messages on Boychat undertaken by Malesky Jr and Ennis (2004) found that over half of posts contained pictures, drawings, poetry, or stories celebrating “boy love” and more than one-fifth of posts validated paedophilic beliefs and abuse of children.

Despite its dubious origins, the phrase “minor attracted” was further popularized by the organization B4U-ACT. Established in 2002 in the United States, B4U-ACT aims to support people who are sexually attracted to children, with the broader social goal of destigmatizing sexual attraction to children (B4U-ACT, 2008). B4U-ACT’s stated mission is to provide pedophiles with information before they act on their sexual impulses, however, the organization’s position is not coherently opposed to child sexual abuse. In fact, B4U-ACT Director of Education Richard Kramer has maintained a website for over 20 years claiming that “[s]ome clinical and many non-clinical studies find the majority of boys are unharmed” by “sexual activity with adults,” and that negative outcomes are more likely when the child is faced with “judgmental adult reactions” to the sexual activity.^3^ That is to say, according to Kramer, the harm of child sexual abuse is attributed to the belief that sexual abuse is harmful, rather than the effect of sexual abuse itself.

In 2007, B4U-ACT used the term “minor-attracted” in a report submitted to the Baltimore Mental Health Systems, with the purported goal of connecting “minor attracted persons” with mental health practitioners (B4U-ACT & TheTogetherChat, 2007). In August 2011, a symposium called “Paedophilia, Minor-Attracted Persons and the DSM” was hosted by B4U-ACT, petitioning for changes within the fifth edition of the DSM around the definition of the word pedophile and pedophilia (B4U-ACT, 2011), and proposing that clinical terminology be altered from pedophilia to “minor-attracted person.” This symposium marks a significant point in the popularization of the term MAPs among those who claim to pursue the goal of sexual abuse prevention, and those who seek to improve the social acceptability of sexual interest in children. Indeed, under the rubric of MAPs, these two goals are often considered interlinked or synonymous.

Goode (2011) described the overarching ideology of “MAPs” activism by drawing on her long-term communication with “Kevin,” a self-identified “minor attracted person” who was publicly campaigning for “MAP” rights. She identified that Kevin saw the world in terms of:

[t]wo opposed camps: the good guys (paedophiles or “minor attracted adults”) on one side; the bad guys (legislators, campaigners and local vigilantes) on the other. Kevin, as a political activist, presents a view of paedophiles as a sexual minority campaigning for rights, in many ways equivalent to the Gay Pride campaigns of the 1970s (Goode, 2011, p. 15).

MAPs activism argues for a clear distinction between pedophilia (viewed as an unchosen sexual orientation) and child sexual abuse (the act of harming a child), which Goode (2011) agrees should be more sharply drawn in clinical, scholarly, and public discourse. However, MAPs activism goes further to propose that pedophiles can and should be “open about their orientation” (Goode, 2011, p. 16) and that this openness should attract no social stigma or concern. The ideological attempt to extend the logic of LGBTIQ+ activism to pedophilia is apparent in the insistence that pedophiles should also be able to come “out of the closet” in a society that accepts them.

In 2012, a key social movement for people sexually attracted to children was established called Virtuous Pedophiles (Virpeds). Virpeds is a peer support organization for pedophiles seeking to support one another not to offend against children. The website Virpeds.org was launched in 2012 and includes a moderated discussion forum for self-identified pedophiles (Nielsen et al., 2020, p. 602). The emergence of Virpeds was of significant interest to those who recognized that online peer support was often the only available support for people who realize they are sexually interested in children (Malone, 2016). A splinter group from Virped emerged in 2016 when “Ender Wiggin” (a pseudonym), took to social media, and in particular Twitter, claiming that sites such as Virpeds do not do enough to destigmatize pedophilia (Weaver, 2018). Wiggins formed an online support group in November 2016 called the “The MAP Support Chat.” The website was taken down but re-grouped and re-named MAP Support Club. Wiggens presented himself online as an anti-offending pedophile however his campaign for the acceptance of pedophilia resulted in repeated bans from Twitter (Weaver, 2018).

The banning of Wiggins and other self-identified “MAPs” on Twitter was opposed by a range of psychologists and anti-abuse organizations, who sent a letter to Twitter advocating for the rights of self-identified pedophiles to use social media to network with one another (Curtis, 2020). During this period of time, a number of child sexual abuse scholars argued that there was a significant population of non-offending pedophiles and this group had been overlooked in research and clinical practice (Levine & Dandamudi, 2016). Cantor and McPhail (2016) agreed that diminishing the gravity of the “experience of stigma associated with having a paedophilic orientation” could be a way forward for treatment. In 2019, Twitter appeared to respond to this expert letter by altering its terms of service to allow people to talk about being pedophiles on the platform (Curtis, 2020).

Wiggins and other self-identified “MAPs” were able to return to Twitter and engage in open discussions about their sexual attraction to children. However, discussions among “MAPs” began to expand to include advocacy for the legalization of cartoon child sexual abuse material, access to child sexual abuse dolls, and, in some cases, the normalization of contact offending (Salter and Hanson, 2021). During this same period, internet safety agencies began reporting significant trading of child sexual abuse material on Twitter (Thalen, 2020). Backlash from child protection advocates and escalating levels of public concern resulted in Twitter changing its terms of service to “clarify” its position on the issue (Curtis, 2020). Salter and Hanson (2021, p. 739) describes Twitter’s policy revision, including a significant expansion of its child sexual exploitation policy to disallow the normalization of pedophilia nor the promotion of it as a sexual orientation.

Up until this point, debate and contestation over the term “minor attracted persons” and their rights to self-representation and promotion of their political agenda had been largely overlooked by the mass media. These issues came to a head in 2021 when the pro-MAP organization Prostasia released a video interview with criminologist Dr Allyn Walker about their book “A Long Dark Shadow: Minor Attracted People and Their Pursuit of Dignity” (Walker, 2021). The book consists of interviews with self-identified “MAPs” and aims to challenge social assumptions about individuals attracted to minors. The release of the interview garnered international media attention after Walker stated that sexual attraction to children is not immoral. They said:

From my perspective, there is no morality or immorality attached to attraction to anyone, because no one can control who they are attracted to at all (Prostasia, 2021).

This claim drew particular ire from conservative and tabloid media, who accused Walker of trying to “normalise paedophilia” (Coulter and Newman, 2021; Fox News, 2021). Following backlash against the interview, Walker stepped down from their position at Old Dominion University at the expiration of their contract (Coulter & Newman, 2021). However, others were in agreement with Walker’s position. One article in the Washington Post by Professor Elizabeth Letourneau and journalist Luke Malone stated that Walker “wasn’t wrong” and that the stigma attached to the word pedophile potentially limits access to support and prevention services (Letourneau & Malone, 2021).

A key factor in the controversy over Walker’s interview was that it was conducted by Prostasia. While describing itself as a “child protection” organization, Prostasia’s campaigns have included advocating for “minor attracted people” to access child sexual abuse dolls and drawn/computer-generated child sexual abuse material. Prostasia’s employees have included convicted sex offenders (Ferdeline, 2020). Additionally, Dr James Cantor, who sits on Prostasia’s advisory council, has openly advocated on social media for the addition of the letter “P” (for pedophile) to be added to the “lesbian, gay, bisexual, transgender, intersex, queer” (LGBTIQ) abbreviation.^4^ Walker’s interview was conducted by Prostasia’s then-Director of Communications, Noah Berlatsky, who has drawn criticism for describing child trafficking as a “moral panic,” referring to sexually exploited children as “young people trading sex” and insisting that police, not child sex offenders, are the main threats faced by sexually exploited children (Emmons, 2021; Wright, 2018). Despite its origins and association with pro-pedophile activism, “minor attracted persons” is a term that continues to surface in scholarly and policy discourse, resulting in media and public backlash (Dearden, 2023). It is therefore timely to consider how this terminology is being deployed in scholarly work.

## Method

The central research question guiding this article is “How is the term ‘minor attracted persons/people’ being applied in the scholarly literature?.” To analyze the literature, a rapid evidence review was undertaken. The review was conducted in January 2024. A systematic search was conducted on the following databases for this inquiry: Criminal Justice Abstracts, ProQuest Social Science, APA PsycArticles, and Violence and Abuse Abstracts. These databases were selected due to their far-reaching collection of psychiatric, social science, and mental health articles. Search terms were “minor attracted persons,” “minor attracted people,” “minor attracted” or “minor-attracted.” To meet the inclusion criteria for the review, the results needed to be peer-reviewed journal articles, in English, and the search terms were to be found within the abstract or title and between the date range of January 2015 to December 2023. Overall, 107 studies were imported into the literature review management software Covidence for screening. After 54 duplicates were removed, 53 studies were screened against title and abstract and 19 studies were excluded at this stage because they were irrelevant to the review. 34 studies were then assessed for full-text eligibility. All three authors screened the studies up to this stage for eligibility using the key questions above to ensure consistency. Four studies were then excluded for a variety of reasons including one being a book chapter, not being in English, a duplicate, and wrong study design. The final review included 30 articles. The full text of all included studies was reviewed and information was collected specifically on the country where the study was conducted, the methods used (including recruitment strategy), how MAPs was defined, information on non-offending MAPs, shame or stigma noted, MAPs as a sexual orientation, child protection implications and limitations of the studies. While rapid evidence reviews are typically focused on summarizing the empirical evidence in an area of scholarship, this research took a thematic approach to understand the construction and use of the term “MAPs” within scholarly literature. The use of the term “MAPs” is still in its infancy, and therefore, the review considers a relatively small number of articles. All articles are in English which potentially limited results from publications in other languages. The review focused on peer-reviewed articles however some book chapters and a book-length manuscript have focused on “minor attracted” people; nonetheless, the authors of these contributions are represented in the peer-reviewed sample of literature included in the review.

## Findings

Among the 30 articles, surveys were the most common research method (*n* = 20) and these were all conducted online. Three of the studies were based on interviews, including two studies with self-identified “MAPs” and one study that recruited journalists to explore how they report on this population. Other articles included evaluations and practice guidance.

### The Definition of MAPs

There was some variation in the definition of the term MAPs in the reviewed papers. It could act as a descriptor for any person with a sexual interest in children, a person meeting the diagnostic criteria for pedophilia or a pedophilic disorder, or a person who self-identifies as a “MAP.” Despite the origin of “MAPs” within pedophile forums and advocacy groups, the term was positioned as a professional and scientific concept. For example, Jackson et al. (2022, p. 317) claimed:

Minor attraction and paedophilia are related terms and are often found overlapping in the scientific literature.

The MAPs term was repeatedly endorsed as the preferred, non-stigmatizing term in comparison to pedophilia. For example, Elchuk et al. (2021, p. 100) stated:

For the purpose of this study, we chose to use the phrase minor-attracted person because it is a common term used by members of the community and is potentially less stigmatizing than clinical language.

In fact, two surveys of people sexually interested in children have found that they prefer medical terms such as “pedophile” and “hebephile” over the term “minor attraction” (Jahnke et al., 2022; Martijn et al., 2020). Nonetheless, the MAPs literature was generally in agreement that they were using the preferred language of such individuals. However, there was a persistent underlying ambiguity within the literature about the relationship between attraction to minors and offending against minors. One article used the term “NOMAPS” (non-offending minor attracted person) to distinguish between non-offending and offending populations (Tenbergen et al., 2021), whereas Levenson et al. (2017, p. 101) appeared to define “MAPs” as synonymous with non-offending or “virtuous paedophiles”:

. . .there are some individuals who refer to themselves as “minor attracted persons” (MAP) or “virtuous paedophiles” who do not act on their attractions. . .

The prospect of non-offending “MAPs” was raised in all articles. The shared narrative across the reviewed papers assumed the existence of a non-offending population alongside, and yet separate from, the offending population, with both groups sexually attracted to children. Ascertaining the size of the population of people sexually attracted to, but not abusing, children is methodologically difficult. Nine of the articles stated that the size of the non-offending population is unknown. Schaefer et al. (2022, p. 392) recognize that:

Identification of MAPs who have never sexually engaged with children is difficult, as many MAPs are fearful to report their attraction to minors due to the stigma associated with this attraction and are fearful of the risk of prosecution.

MAPs scholarship has sought to recruit self-identified, non-offending MAPs for study, often circulating research information through online networks and discussion boards dedicated to people sexually interested in children, or more general social media recruitment strategies. Lievesley and Lapworth (2022, p. 880) acknowledged the complexity of obtaining a sample of the non-offending population, given that “offending status cannot be completely eliminated” when researchers are recruiting “MAPs.” In the study by Stevens and Wood (2019), men were categorized as “non-offending” if they were not “currently” abusing children. As a result, their sample of “non-offending” MAPs included men with criminal convictions for child sex offenses. Stevens and Wood (2019, p. 970) explained:

This study represents one of the initial investigations of a non-offending MAP population, however, although at interview the sample were dedicated to not offending several participants had historical convictions, confounding the sample. . .

In summary, there was nuanced variation in the application of the term MAPs, which was used to describe overlapping phenomena, including sexual interest in children, distinct from or commensurate with offending, as well as a self-identity or a label. The latter use of the term MAPs was expanded upon in characterizations of this group as an oppressed sexual minority, which will be explored in the next section.

### MAPs as an Oppressed Sexual Minority

The proposition that sexual attraction to minors constitutes a sexual orientation or identity (rather than a paraphilia or psychopathology, as outlined in psychiatric diagnostic manuals) and that MAPS are therefore a sexual minority, was presented by the majority of articles (*n* = 18). In some cases, researchers suggested that their research participants themselves framed minor attraction as a sexual orientation. As stated by Grady et al. (2019, p. 406):

Some participants indicated that their attractions should be understood as a form of sexual orientation.

In other publications, scholars were explicit that they categorized sexual interest in children as “queer” or otherwise similar and comparable to the sexual interests of LGBTIQ+ people. This position was most strongly asserted by Walker and Panfil (2016, p. 40) who stated:

. . .we compare the experiences and statuses of “MAPs” to other queer populations (specifically, LGBT individuals) in order to illustrate critical similarities between these groups.

Once sexual interest in children had been designated as “queer,” Walker and Panfil (2016, p. 49) asserted that sexual interest in children should be destigmatized, in the same way that same-sex attraction or gender diversity should be destigmatized.

As with other queer populations, it is important to shed light on the lives of minor-attracted persons in ways that will diminish societal stigma toward this population: while MAPs are currently thought of as predatory threats to children, research using a deconstructionist framework can discourage the idea of the essentialized identities of MAPs to reveal the complexities within their lives.

This excerpt presents two points of confusion. Firstly, while it seeks to “discourage the idea of the essentialized identities of MAPs,” particularly where sexual interest in children is conflated with the sexual abuse of children, it simultaneously positions “minor attraction” as a sexual identity, comparable to identifying as gay, lesbian or trans. This passage is not rejecting the notion of an essentialized identity, but instead advocates for the substitution of one identity based on medical notions of pedophilia for another based on “queer” sexual interests. Secondly, by comparing pedophilia to “queer” sexual attraction, it conflates arousal to non-consensual sexual activity (that is, the sexual abuse of children) with arousal to consensual sex between adults.

Such parallels between sexual interest in children, and same-sex attraction, have long been rejected by LGBTIQ+ organizations and communities, and such comparisons are generally agreed to be discriminatory against LGBTIQ+ people. However, the MAPs literature often assumed a parallel or corollary between sexual interest in children and same-sex attraction. For instance, Levenson and Grady (2019, p. 382) described a process whereby negative views of pedophilia are internalized by MAPs in harmful ways that closely mirror descriptions of internalized homophobia:

Unsurprisingly, these [negative] societal responses are adopted by MAPs, infusing psychologically damaging beliefs into their own identity. . ..

Even where a comparison with LGBTIQ+ people was not explicit, the proposition that sexual attraction to children is the legitimate basis of a sexual identity framed MAPs as an oppressed sexual minority. MAPs scholarship often advocated for the development of a positive MAPs self-identity in ways that appear contrary to aims of treatment programs for men sexually interested in children, which generally promote identities based on normative interests and activities rather than sexual proclivity (Richards, 2021). This point was recognized by Lievesley and Harper (2022, p. 3), who recommended that:

Having made the decision to change, an individual with sexual convictions should then engage with treatment to allow them to work towards their possible positive future identity.

Despite this cautionary note, the MAPs literature tended to assume that sexual interests should or would constitute a self-identity or group identity for people sexually aroused by children. Once sexual interest in children was categorized as a sexual orientation and identity, MAPs scholarship made a number of consequential assertions about legal and social reforms.

### Stigma and Pro-MAP Reform

Stigma was the major theme in the MAPs scholarship and was referred to within all reviewed articles. Stigma was described in a number of ways: as a barrier for pedophiles seeking to accessing services, as a barrier to effective practice among clinicians treating this population, as a risk factor for offending, and as a form of social oppression and undue discrimination against individuals with a sexual interest in children. These forms of stigma were often presented as interconnected. For instance, a persistent claim throughout the literature was that social norms against sexual interest in children increased the risk of offending against children because these social norms are a barrier against help-seeking, and they also negatively impacted MAPs’ mental health, making it more likely that they would act on their sexual interests. Jara and Jeglic (2021, p. 308) state:

. . .stigma and lack of social acceptance from the public toward MAPs serve as barriers to the treatment of MAPs and child sexual abuse prevention policies. They also add to the risk factors which may lead to increased child sexual offending.

A contrary view was put forward by Levenson et al. (2019, p. 384), who recognized that stigma may be a motivator for help-seeking:

. . . “MAPs” themselves have identified a range of other psychological needs that are important to them when seeking counselling. . .These goals include a desire to improve self-esteem, decrease isolation, and deal with the socio-cultural stigma of minor attraction. (references removed).

References to “stigma” in the MAPs literature included acknowledgment that pedophiles can be stigmatized by clinicians when they seek help, and that clinicians needed to understand the impact of stigma on the mental health of people sexually interested in children (e.g., Cohen et al., 2020). These discussions included the fraught issue of mandatory reporting obligations for therapists with a client disclosing sexual interest in children. Commensurate with their focus on non-offending people sexually interested in children, the MAPs scholarship generally recommended that, absent other risk factors or evidence, professionals should not make such a mandatory report. Lievesley and Harper (2022, p. 8) argue that:

. . .if a professional conflates minor attraction with child sexual abuse, it quickly becomes a reasonable suspicion that a MAP who seeks treatment could be a risk to children that s/he has regular contact with. This is even the case in the absence of any objective evidence of abuse, as some would (often incorrectly) infer that attraction is an indicator of imminent sexual offense risk.

A lack of professional and legal guidance around mandatory reporting obligations when working with people disclosing sexual interest in children remains an ongoing barrier to service delivery with this population. However, some MAPs scholars presented an emphatic case that professional views that people sexually interested in children are more likely to abuse children constituted a form of stigma and discrimination. Walker et al. (2019, 2022) repeatedly claimed that the belief that “minor attracted” people are a risk to children is false and discriminatory, and comparable to the belief that gay men are pedophiles. A recent paper stated that a client sexually interested in children should only be reported to authorities if they indicate that they have, or intend, to abuse a child (Walker et al., 2022).

For some authors, the stigmatization of sexual interest in children was a primary driver of child sexual abuse. Lievesley and Harper (2022, p. 9) state:

At the core of addressing the social context of prevention is understanding and tackling social stigma about minor attraction.

In all, 10 of the articles called for the de-stigmatization or at least a reduction of stigma against pedophilia as itself a barrier to treatment. For example, McKillop and Price (2023, p. 697) state:

Combined, it seems that dispelling the stigma associated with minor attraction toward children and MAPs and to identify ways to break down barriers to providing (and seeking) support, is an essential component of current (and future) preventative action.

These recommendations are contrary to the long-standing recognition that norms against child sexual abuse have a deterrent and preventative effect. For instance, Finkelhor (2008, p. 9) stated that societal “norms” play a vital role in crime reduction and that, when “norms are clear and strict, offenses are discouraged.” In his foundational work on motivations for child sex offending, Finkelhor (1984) outlines that child sexual abusers have to overcome internal and external inhibitions against child sexual abuse in order to harm children. In this regard, stigma is both an internal and external inhibiter whereby a potential offender is restricted by social rules and punitive consequences that are socially enforced and individually internalized (Arneson, 2007). However, only one article recognized the role of stigma in delineating between acceptable and unacceptable behavior. Parr and Pearson (2019, p. 948) explained:

Of course, stigma may be a useful guide to normative behaviour. Rather than a barrier, it could be viewed as a behavioural guide, aiding our understanding of what is and is not “acceptable” behaviour in our environment.

Lievesley et al. (2023) provided a more critical perspective on the claims of MAPs that they are burdened with shame and stigma. The authors recognized that research participants may have a “self-presentation bias” in their prioritization of stigma as a mental health concern, over and above other clinical considerations, such as their risk to children. They state:

. . .the MAPs in our sample may have over-prioritized mental health and stigma-related treatment targets and downplayed the extent to which they require support with managing their sexual attractions.

This was one of the few references in the reviewed literature to the possibly self-interested responses of men with a sexual interest in children, who may present an overly sympathetic picture of themselves as unduly stigmatized while downplaying their risk to children. Writings by Walker and colleagues went further to claim that, in the absence of stigma and other aggravating factors, “MAPs” pose no specific risk to children. For instance, Walker and Panfil (2016, p. 47) claim that a “primary attraction to minors” has no “causal relationship” with offending against minors in the absence of other risks:

We seek to highlight the fact that primary attraction to minors does not appear to have a clear causal relationship to committing person offenses against children, in that minor attraction by itself seems neither necessary nor sufficient to explain interpersonal forms of sexual offending.

The possibility that sexual attraction to children could pose a risk to children was further challenged by Walker et al. (2022, p. 54) in the following claim:

It may be that the majority of individuals who commit sex offenses against children do so for opportunistic reasons rather than because they are preferentially attracted to them.

The contradictory position that “MAPs” are unfairly stigmatized as a risk to children, but that they pose more of a risk to children because of this stigma, recurred throughout the “MAPs” literature. The effect of this argument was to displace the threat to children from people with a sexual interest in children to social and cultural attitudes who consider such individuals to be a risk to children. The role of stigma in preventing or inhibiting abuse was ignored by all but one author and no discussion was offered regarding the consequences in the event that the stigma of sexually abusing children is removed.

## Discussion

This review has examined the ways in which the term “MAPs” is used in recent scholarly literature. Overall, there was a lack of consensus on the definition of the term, which was sometimes presented as a less stigmatizing synonym for pedophilia, but also as a way to refer to a sexual identity, orientation or minority. These variations were conceptually confusing, particularly when used as an overall frame for research based on samples of self-identified “MAPs” where the boundaries between offenders and non-offenders were indistinct. Attempts to recruit “non-offending” pedophiles for research purposes are confounded by the fact that it is not possible to ascertain whether an individual has, or has not, offended against a child (Stevens & Wood, 2019, p. 970), a matter about which child sex offenders routinely lie (Bourke et al., 2015). Nonetheless, this review found that the term “MAPs” has gained a level of legitimacy within scholarly literature and utilized as common parlance among some authors. No author disputed the use of either “minor attracted people/person” or “MAPs,” but they generally agreed that the term “MAPs” is less stigmatizing than “pedophile” and is the preferred term among people sexually attracted to minors.

As previously explained, this assumption has been proven incorrect in light of recent surveys which found that pedophiles prefer to self-identify as “paedophiles” rather than “minor attracted.” It is also self-evident from the explosive public backlash whenever the MAPs term is used in policy or public debate that the term is not less stigmatized than “pedophile.” It is therefore curious why these scholars have embraced the term since their primary rationale for its use is not supported. One explanation is the collaborative relationships evident between these scholars and online communities and organizations that are promoting the term MAPs. For the most part, MAPs scholars are recruiting research participants from online communities and organizations for pedophiles, and these groups are united by the shared goal of destigmatizing sexual interest in children, which they pursue under the rubric of “MAPs.” Earlier work by Goode (2009) analyzed how the personal reflections of self-identified MAPs had shaped by the conflicted cultural norms and values of online pedophile groups, however her iterative and reflective approach was largely absent in the reviewed articles. The development and promotion of the MAPs term by online pedophile communities were elided in MAPs scholarship. Nor did MAPs scholarship interrogate the fact that they are interviewing and surveying members of social movements with ambivalent, positive, or dissembling views about the sexual abuse of children.

The reviewed literature largely agreed that “MAPs” are an unfairly stigmatized sexual minority. The treatment literature has long recognized the impact of stigma on child sex offenders. For instance, in her work on post-release support for serious child sex offenders, Richards (2021) argues that addressing stigma on an individual level helps people form a new identity based on social connection and relationships, which can bring about behavioral changes and ultimately promote desistance from sexually offending. However, the distinction between stigma informed treatment, and social stigma, was conflated in the MAPs scholarship, such that clinical recommendations for addressing stigma in treatment contexts were generalized to make the case for broader social and cultural reform. Many MAPs scholars evinced a strong opposition to any stigmatization of sexual interest in children, which was described as unfounded, harmful, and discriminatory. However, contrary to claims that “stigma” is the primary driver of child sexual abuse, institutions and environments that have “de-stigmatized” sexual interest in children have been places of rampant sexual abuse and exploitation (e.g., Clegg, 2021). At the very least, such counter-examples should temper the confidence of MAPs researchers that reducing the stigmatization of pedophilia will reduce rates of child sexual abuse.

The MAPS literature assumed a parallel between pedophilia and same-sex attraction to the point where research on the minority stress experienced by gays and lesbians was regularly cited to justify the claim that pedophiles are unduly harmed by the social stigmatization of child sexual abuse. However, it is unclear that “MAPs” are a sexual minority in any meaningful sense. A nationally representative survey of almost 2000 Australian men found that one in six expressed some sexual interest in children and young people under the age of 18 (Salter et al., 2023), which suggests that sexual interest in minors is relatively common among men in the community. This research finds a strong overlap between sexual interest in children and other deviant sexual interests, including bestiality and sadism (Salter et al., 2023), which calls into question characterizations of pedophilia as a normative sexual orientation or identity. In this study, one in three men sexually interested in children had committed a child sex offense, online and/or offline, compared to one in twenty-five men who were not sexually interested in children (Salter et al., 2023). Hence, the widely held belief that people with a sexual interest in children are a risk to children is rational. Characterizations of this belief as discriminatory trivializes the threat to children posed by paedophilic sexual interest.

A recurrent implication within MAPs scholarship is that child sexual abuse perpetration by pedophiles is driven, to a significant degree, by the stigmatization of pedophilia. A proportion of child sex offenses were explained in the reviewed papers as the behavior of an oppressed sexual minority denied access to necessary mental health care and driven to abuse children by shame and stigma. It is undoubtedly true that the availability of early intervention services for pedophiles have been curtailed by disgust at child sexual abuse, and these services have an important role to play in prevention responses (Austin & Salter, 2023). However, the framing of child sex offenses as the “acting out” of oppressed pedophiles is not only an extraordinarily sympathetic account of sexual violence against children, but it is a poor fit with the forensic record, which has documented the prevalence of premeditated, manipulative grooming and other deliberative patterns of behavior with a systematic focus on overcoming barriers to abuse and enforcing obedience and silence in children (e.g., Winters & Jeglic, 2017). The displacement of responsibility for abuse from those who commit it, to those who abjure it, is an unusual feature of MAPs scholarship, and it is difficult to find parallel examples in other areas of prevention scholarship. For example, although domestic violence is common, it is a stigmatized behavior. Accordingly, men who experience the impulse to victimize an intimate partner typically keep these feelings secret, and experience significant shame (Gadd, 2002). While efforts to prevent violence against women include efforts to engage early with such men, domestic violence prevention does not promote the “destigmatization” of the impulse to physical or sexual violence. To the contrary, public health and social marketing campaigns have sought to reinforce social norms against the acceptability of domestic violence (Jewkes et al., 2015).

MAPS scholarship did not present a coherent agenda for the “destigmatization” of pedophilia. Few would disagree with the suggestion that media coverage of pedophilia and child sexual abuse should be nuanced and sensitive. Ischebeck et al. (2021) interviewed journalists to explore their knowledge and attitudes toward sexual interest in children, and found that their respondents were well informed about pedophilia, and held balanced views about people with a sexual interest in children. Their study does not support one-dimensional characterizations of the media and cultural landscape as entirely hostile toward pedophiles. Attempts by MAPs scholars to evaluate interventions designed to destigmatize sexual interest in children and change public attitudes have found that these efforts are ineffective and can result in an *increase* in negative attitudes (Jara & Jeglic, 2021; McKillop & Price, 2023). Furthermore, evidence suggests it is possible to provide non-judgmental and meaningful treatment for pedophiles without pursuing the “de-stigmatisation” of pedophilia within a given social or cultural context. Examples can be drawn from the service Stop It Now! in the United Kingdom, which receives a high level of service engagement and interest each year from people concerned about their sexual feelings and behavior toward children, within a social context in which paedopihlia remains stigmatized (Grant et al., 2019). Interestingly, and relevant to the use of the term ”MAPs,” the same program emphasizes the importance of using correct terminology on the subject of child sexual abuse and pedophilia, rather than “hiding” behind alternative language and euphemisms (Hudson, 2017, p. 105).

## Conclusion

Research with undetected child sex offenders and men in the community with a sexual interest in children provides an important evidence base for prevention approaches. However, organizations and networks of self-identified pedophiles offers a fraught opportunity for such research, since pedophiles can use those interactions with researchers to promote their own aims and objectives (Goode, 2009). As this review has shown, the transposition of the terminology of MAPs from online pedophile networks to the academic community has brought with it a questionable set of commitments, including comparisons between pedophiles and oppressed sexual minorities, and the recommendation that sexual interest in children is “de-stigmatised.” Such conclusions do not originate within the academic or scientific literature, but were first formulated by the pro-pedophile advocacy movement, which predates MAPs scholarship by several decades. It should not be a surprise that scholars who source their research participants from such organizations will find their data is skewed toward the agenda of those organizations, however, this likelihood was rarely countenanced in the MAPs scholarship.

The findings of this review caution against the unproblematic adoption of the terminology and argumentation of pro-pedophile groups within child sexual abuse scholarship. MAPS research is situated within a longer history of research with undetected pedophiles in the community that, in the absence of critical distance, risks laundering the political agendas of pro-pedophile groups as “scientific” recommendations. The overwhelming focus on anti-pedophile stigma and shame in the MAPS scholarship may reflect the personal biases of their research subjects and collaborating organizations. Potentially, this includes the common cognitive distortion among child sex abusers is that the primary harm of sexual abuse to children comes from the stigma that attaches to abuse, rather than the conduct of the abusers. Academic researchers should be very careful to ensure that such distortions are not being introduced and inadvertently amplified in their engagement with self-identified MAPs. Anonymous online surveys distributed to all community members have proven to be an effective way of studying sexual interest and offending against children among undetected offenders without mediation or intervention by pro-pedophile networks (Salter et al., 2023), and research into the MAPs phenomenon should consider the broader subcultural dynamics of pro-pedophile movements and their impacts on the individual beliefs, identities and arguments of pedophiles (Goode, 2009, 2011).

Much of the argumentation contained in the MAPs scholarship rested on an assumed similarity between pedophilia and same-sex attraction. We close by emphasizing that these comparisons have been consequential. Controversies over academic use of the term MAPs have had a role to play within right-wing anti-LGBTIQ+ movements, including the claim that LGBTIQ+ people are “grooming” and sexualizing children (Shah, 2023). Such despicable accusations have been fueled by the confident assertions of some MAPs scholars that pedophiles are “queer” or otherwise belong under the LGBTIQ+ umbrella. The opportunistic right-wing capitalization on such claims illustrates the irresponsibility of academic parallels between pedophilia and same-sex attraction, which not only undermines and delegitimizes the project of sexual abuse prevention, but it is also feeding into politically extremist rhetoric against LGBTIQ+ people.

## Supplemental Material

sj-docx-1-tva-10.1177_15248380241270028 – Supplemental material for A Review of Academic Use of the Term “Minor Attracted Persons” Supplemental material, sj-docx-1-tva-10.1177_15248380241270028 for A Review of Academic Use of the Term “Minor Attracted Persons” by Christina Farmer, Michael Salter and Delanie Woodlock in Trauma, Violence, & Abuse

## Notes

1. This article uses the term “pedophile” to refer to adults who have an enduring and persistent sexual interest in children. Psychiatric debates over the definitions and distinctions between “paedophilia,” “hebephilia,” and sexual interest in children are ongoing and beyond the scope of this article. While we acknowledge that not all individuals with a sexual interest in children meet the diagnostic criteria for a paedophilic disorder, we use this terminology because the article is referring to individuals and groups who evince a significant level of sexual interest in children, to the point where they are motivated to identify as pedophiles to themselves and others, to socialize with other pedophiles online, to seek help for their sexual feelings and/or to pursue social and legal reform to accommodate the sexual abuse of children.

2. Recent examples that have raised public ire include a notorious paper in which the researcher described himself masturbating to drawn child sexual abuse material (Batty, 2022), as well as papers that compared ejaculating on a child to ejaculating on a shoe (Breslow, 2011) and legitimizing sexual activity between family members (Thorneycroft, 2019).

3. https://www.mhamic.org/myths/harm.htm.

4. https://archive.ph/GzvTC.
