# Supplementary material for: A Review of Academic Use of the Term “Minor Attracted Persons”
Source: Trauma Violence Abuse. 2024 Sep 15;25(5):4078–89. doi: 10.1177/15248380241270028 (PMC11545205; doi:10.1177/15248380241270028)
Supplement: sj-docx-1-tva-10.1177_15248380241270028 – Supplemental material for A Review of Academic Use of the Term “Minor Attracted Persons” [file sj-docx-1-tva-10.1177_15248380241270028.docx]

**MAPs Summary of studies**

| **Study** | **Location** | **Research method** | **Objective of study** | **Major findings** |
| --- | --- | --- | --- | --- |
| Cohen, L. J., Ndukwe, N., Siegfried, R., Kopeykina, I., Yaseen, Z. S., & Galynker, I. (2018). Attraction versus action in pedophilic desire: the role of personality traits and childhood experience. *Journal of Psychiatric Practice*, 24(6), 374-387. | Multi-country | Mixed methods | The study compares pedophiles who abstain from child sexual abuse with those who do not seeking to understand factors differentiating attraction from behavior, aiding prevention efforts. | Antisocial traits drive pedophilic behavior apart from attraction. Social inhibition and cognitive distortions correlate with attraction; causation unclear. Childhood sexual abuse increases risks for both attraction and behavior. |
| Cohen, L. J., Wilman-Depena, S., Barzilay, S., Hawes, M., Yaseen, Z., & Galynker, I. (2020). Correlates of chronic suicidal ideation among community-based minor-attracted persons. *Sexual Abuse*, 32(3), 273-300. | Multi-country | Online survey | This study aims to investigate elevated suicidal risk in adults attracted to minors. | 38.1% endorsed chronic suicidal ideation, unrelated to child sexual engagement or justice system contact. Various risk factors identified. |
| Cohen, L., Ndukwe, N., Yaseen, Z., & Galynker, I. (2018). Comparison of self-identified minor-attracted persons who have and have not successfully refrained from sexual activity with children. *Journal of Sex & Marital Therapy*, 44(3), 217-230. | Multi-country | Online survey | This study aims to investigate differences between adults who are sexually attracted to children with and without histories of child sexual activity to identify protective and risk factors. | Study reveals significant differences between adults with and without histories of child sexual activity, including age, traits, and attitudes. |
| Elchuk, D. L., McPhail, I. V., & Olver, M. E. (2022). Stigma-related stress, complex correlates of disclosure, mental health, and loneliness in minor-attracted people. *Stigma and Health*, 7(1), 100. | Multi-country | Online survey | To investigate stigma-related stressors' impact on psychological distress in those attracted to children, examining relationships with relational quality, loneliness, and suicidality. | Study reveals perceived lack of support and loneliness correlate with psychological distress and suicidality among people attracted to children. |
| Grady, M. D., & Levenson, J. S. (2021). Prevalence rates of adverse childhood experiences in a sample of minor-attracted persons: A comparison study. *Traumatology*, 27(2), 227. | Multi-country | Online survey | Explore adverse childhood experiences (ACEs) in males sexually attracted to children and compare to general male population and individuals convicted of sexual offenses. | Men sexually attracted to children experienced higher rates of ACEs compared to the general male population, emphasizing the importance of trauma-informed approaches in prevention efforts. |
| Grady, M. D., Levenson, J. S., Mesias, G., Kavanagh, S., & Charles, J. (2019). “I can’t talk about that”: Stigma and fear as barriers to preventive services for minor-attracted persons. *Stigma and Health*, 4(4), 400. | Multi-country | Online survey | Investigate treatment-seeking experiences of those sexually attracted to children through qualitative analysis, focusing on themes such as stigma, shame, and community support. | Stigma significantly influences the willingness to seek treatment. |
| Harper, C. A., & Lievesley, R. (2022). Exploring the ownership of child-like sex dolls. *Archives of Sexual Behavior*, 51(8), 4141-4156. | Multi-country | Online survey | Investigate psychological characteristics and sexual aggression proclivities of child-like sex doll owners versus non-owners among those sexually attracted to children. | Child-like sex doll owners show lower sexual preoccupation but higher sexually objectifying behaviors and anticipated enjoyment of child encounters. |
| Iffland, J. A., & Schmidt, A. F. (2023). Stigmatization and perceived dangerousness for intrafamilial child sexual abuse of fathers with a history of sexual offenses and paraphilic interests: results from a survey of legal psychological experts. *Child Abuse & Neglect,* 144, 106348. | Germany | Online survey | Evaluate legal psychological experts' perspectives on intrafamilial child sexual abuse risk assessment, contact limitations, and stigmatization of those sexually attracted to children. | Experts view fathers/stepfathers with diagnosed pedophilic disorder as most dangerous, supporting complete contact bans; also observe reduced parental capacity with sexual offending history. |
| Ischebeck, J., Kuhle, L. F., Rosenbach, C., & Stelzmann, D. (2021). Journalism and pedophilia: Background on the media coverage of a stigmatized minority. *Stigma and Health*, 9(1), 20–29. | Germany | Interviews | Investigate journalists' knowledge, attitudes, and emotions towards pedophilia, exploring their role in destigmatization and prevention of child sexual abuse. | Journalists generally showed accurate knowledge of pedophilia but overestimated the risk of those sexually attracted to children becoming offenders, highlighting misinformation. |
| Jackson, T., Ahuja, K., & Tenbergen, G. (2022). Challenges and solutions to implementing a community-based wellness program for non-offending minor attracted persons. *Journal of Child Sexual Abuse*, 31(3), 316-332. | United States | Evaluation | Address barriers to providing preventive services to non-offending people sexually attracted to children in the United States and propose solutions. | Identifies barriers to preventive services including concerns about mandated reporting, stigmatization, and therapist knowledge gaps, offering solutions. |
| Jahnke, S., Blagden, N., & Hill, L. (2022). Pedophile, Child Lover, or Minor-Attracted Person? Attitudes Toward Labels Among People Who are Sexually Attracted to Children. *Archives of Sexual Behavior*, 51(8), 4125-4139. | Multi-country | Online survey | Investigate attitudes towards labels for individuals attracted to children, aiming to understand preferences and challenges in reducing stigma. | Study finds acceptance of "pedophile/hebephile" and "minor-attracted person" labels, highlighting challenges in finding non-stigmatizing terminology. |
| Jara, G. A., & Jeglic, E. (2021). Changing public attitudes toward minor attracted persons: An evaluation of an anti-stigma intervention. *Journal of Sexual Aggression*, 27(3), 299-312. | Multi-country | Online survey | Investigate public attitudes towards those sexually attracted to children and assess the impact of a psychoeducational text on attitudes to reduce stigma. | Reading a psychoeducational text on those sexually attracted to children was associated with more negative attitudes, posing challenges for developing anti-stigma interventions. |
| Levenson, J. S., & Grady, M. D. (2019). “I could never work with those people...”: Secondary prevention of child sexual abuse via a brief training for therapists about pedophilia. *Journal of Interpersonal Violence*, 34(20), 4281-4302. | United States | Evaluation | Develop and assess a training workshop for clinical therapists to ethically and effectively counsel individuals with pedophilic interests. | Training workshop led to improved knowledge and attitudes among therapists, enhancing willingness and perceived competence to work with those sexually attracted to children. |
| Levenson, J. S., & Grady, M. D. (2019). Preventing sexual abuse: Perspectives of minor-attracted persons about seeking help. *Sexual Abuse*, 31(8), 991-1013. | Multi-country | Online survey | Explore people sexually attracted to children’s experiences with help-seeking, barriers to seeking assistance, and treatment priorities to inform counselling and preventive interventions. | Most people sexually attracted to children sought professional help, but barriers included confidentiality concerns and therapist knowledge gaps. Treatment priorities included mental health and reducing attraction to minors. |
| Levenson, J. S., Grady, M. D., & Morin, J. W. (2020). Beyond the “ick factor”: Counseling non-offending persons with pedophilia. *Clinical Social Work Journal*, 48, 380-388. | United States | Practice guidelines | Explore how mental health professionals can provide effective, ethical, and compassionate services for non-offending people attracted to children to prevent sexual abuse. | Understanding treatment needs and overcoming obstacles to help-seeking are crucial for clinicians to contribute to sexual abuse prevention. |
| Lievesley, R., & Harper, C. A. (2022). Applying desistance principles to improve wellbeing and prevent child sexual abuse among minor-attracted persons. *Journal of Sexual Aggression,* 28(1), 1-14. | N/A | Policy framework | Propose an extension of desistance theory to provide a theoretical framework for preventing sexual abuse among those sexually attracted to children. | Extending desistance theory offers a framework for prevention initiatives addressing mental health and the prevention of sexual offending. |
| Lievesley, R., & Lapworth, R. (2022). “We Do Exist”: The Experiences of Women Living with a Sexual Interest in Minors. *Archives of Sexual Behavior*, 1-18. | United Kingdom and United States | Interviews | Investigate the experiences of female’s who are sexually attracted to children to address the gap in literature focusing predominantly on male experiences. | Semi-structured interviews reveal unique challenges faced by female’s sexually attracted to children, emphasizing social isolation and identity impacts, offering insights for service provision. |
| Lievesley, R., Harper, C. A., & Elliott, H. (2020). The internalization of social stigma among minor-attracted persons: *Implications for treatment. Archives of Sexual Behavior*, 49(4), 1291-1304. | Multi-country | Online survey | Investigate how internalized stigma among those sexually attracted to children affects help-seeking behaviors and avoidance of children. | Higher levels of thought suppression and lower psychological well-being among those sexually attracted to children are associated with increased shame, guilt, and avoidance of children. |
| Lievesley, R., Harper, C. A., Swaby, H., & Woodward, E. (2023). Identifying and working with appropriate treatment targets with people who are sexually attracted to children. *Journal of Sex & Marital Therapy*, 49(5), 497-516. | Multi-country | Online survey | Investigate treatment goal prioritization among those sexually attracted to children and predictors thereof to improve professional practices working with this population. | Self-compassion drives treatment goal prioritization among people sexually attracted to children, highlighting the importance of collaborative and effective approaches in professional practice. |
| Lievesley, R., Swaby, H., Harper, C. A., & Woodward, E. (2022). Primary health professionals’ beliefs, experiences, and willingness to treat minor-attracted persons. *Archives of Sexual Behavior*, 51(2), 923-943. | United Kingdom, United States, Canada, Australia, and New  Zealand | Online survey | Investigate non-specialist healthcare providers' beliefs, knowledge, and decision-making processes when working with people disclosing sexual attractions to children. | Primary medical and mental health professionals differ in views on dangerousness and control over behaviors; training is needed to improve comfort working with those sexually attracted to children. |
| McKillop, N., & Price, S. (2023). The Potential for Anti-Stigma Interventions to Change Public Attitudes Toward Minor-Attracted Persons: A Replication and Extension of Jara and Jeglic’s Study. *Journal of Child Sexual Abuse*, 1-21. | Australia | Online survey | Test the effectiveness of different educational messaging modes in reducing negative attitudes toward those sexually attracted to children in Australia. | Educational interventions led to reduced negative attitudes toward those who are sexually attracted to children overall, with no significant differences between messaging modes; older participants held less negative attitudes. |
| Parr, J., & Pearson, D. (2019). Non-offending minor-attracted persons: Professional practitioners’ views on the barriers to seeking and receiving their help. *Journal of Child Sexual Abuse*, 28(8), 945-967. | United Kingdom | Online survey | Investigate barriers faced by non-offending people sexually attracted to children in seeking help and explore professionals' perspectives on reducing these barriers. | Accessibility of treatment and perceived risk of disclosure emerged as main barriers; suggested solutions include increased publicity, public education, and enhanced professional training. |
| Schaefer, A., Wittenberg, A., Galynker, I., & Cohen, L. J. (2023). Qualitative Analysis of Minor Attracted Persons’ Subjective Experience: Implications for Treatment. *Journal of Sex & Marital Therapy*, 49(4), 391-411. | Multi-country | Online survey | Explore experiences and perspectives of those who are attracted to children regarding treatment accessibility and societal understanding through thematic analysis of narrative responses. | Participants emphasized distinctions between attraction and action, highlighted the immutability of being sexually attracted to children, and stressed the need for therapy to reduce distress. |
| Schmidt, A. F., & Niehaus, S. (2022). Outpatient therapists’ perspectives on working with persons who are sexually interested in minors. *Archives of Sexual Behavior*, 51(8), 4157-4178. | Switzerland | Online survey | Explore outpatient therapists' attitudes toward individuals sexually interested in children and their willingness to provide treatment. | Therapists showed diverse levels of stigma towards individuals sexually interested in children, impacting their readiness to treat and perceived competency. |
| Spriggs, S. A., Cohen, L. J., Valencia, A., Yaseen, Z. S., & Galynker, I. I. (2018). Qualitative analysis of attitudes toward adult-child sexual activity among minor-attracted persons. *Journal of Sex & Marital Therapy*, 44(8), 787-799. | Multi-country | Online survey | Explore perspectives from those sexually attracted to children on the effects of adult-child sexual interactions to inform clinical approaches and treatment efficacy. | Narrative responses from self-identified people sexually attracted to children revealed diverse perceptions of harm and mitigating/aggravating factors, informing clinical practice. |
| Stevens, E., & Wood, J. (2019). “I despise myself for thinking about them.” A thematic analysis of the mental health implications and employed coping mechanisms of self-reported non-offending minor attracted persons. *Journal of Child Sexual Abuse,* 28(8), 968-989. | Multi-country | Content analysis | Explore coping mechanisms and mental illness among non-offending pedophiles to inform rehabilitation and support strategies. | Thematic analysis of forum posts (n=5,210) revealed coping strategies including risk management and mood regulation, alongside prevalent mental health issues. |
| Tenbergen, G., Martinez-Dettamanti, M., & Christiansen, C. (2021). Can nonoffending pedophiles be reached for the primary prevention of child sexual abuse by addressing nonoffending individuals who are attracted to minors in the United States? New Strategies With The Global Prevention Project. *Journal of Psychiatric Practice*, 27(4), 265-272. | United States | Review | Introduce a novel prevention strategy, The Global Prevention Project, for nonoffending people sexually attracted to children in the U.S., addressing legal, treatment, and implementation challenges. | Outlined a comprehensive prevention strategy targeting people attracted to children, emphasizing treatment transparency, legal considerations, and implementation challenges, with proposed solutions. |
| Walker, A. (2019). “I’m not like that, so am I gay?” The use of queer-spectrum identity labels among minor-attracted people. *Journal of Homosexuality.* 67(12), 1736–1759. | United States | Interviews | Investigate the use of queer-spectrum identity labels by people sexually attracted to children and the conflicts they face. | People sexually attracted to children face conflicts over the use of queer-spectrum identity labels, challenging assumptions about sexual orientation and highlighting the complexities of identity. |
| Walker, A., & Panfil, V. R. (2017). Minor attraction: A queer criminological issue. *Critical Criminology*, 25, 37-53. | N/A | N/A | Challenge societal suspicion and stigma towards those sexually attracted to children by employing queer criminology, exploring parallels with the LGBT community and implications for criminal justice. | The prevailing assumption of pedophiles as mentally ill and predatory warrants re-evaluation through queer criminology, offering insights for more nuanced criminal justice approaches. |
| Walker, A., Butters, R. P., & Nichols, E. (2022). “I would report it even if they have not committed anything”: Social service students’ attitudes toward minor-attracted people. *Sexual Abuse*, 34(1), 52-77. | United States | Online survey | Investigate mental health students' perceptions of those sexually attracted to children focusing on their beliefs about reporting obligations and confidentiality. | Over half of surveyed students believed pedophiles must be reported to police, regardless of offense history. Students lack understanding of reporting guidelines. |
